# Sepsis-Associated Acute Kidney Injury: What’s New Regarding Its Diagnostics and Therapeutics?

**DOI:** 10.3390/diagnostics14242845

**Published:** 2024-12-17

**Authors:** Dimitris Kounatidis, Ilektra Tzivaki, Stavroula Daskalopoulou, Anna Daskou, Andreas Adamou, Anastasia Rigatou, Evangelos Sdogkos, Irene Karampela, Maria Dalamaga, Natalia G. Vallianou

**Affiliations:** 1 Diabetes Center, First Department of Propaedeutic Internal Medicine, Laiko General Hospital, Medical School, National and Kapodistrian University of Athens, 11527 Athens, Greece; dimitriskounatidis82@outlook.com; 2 First Department of Internal Medicine, Sismanogleio General Hospital, 15126 Athens, Greece; ilektra.papagianni@gmail.com (I.T.); daskouanna@gmail.com (A.D.); aadamou@gmail.com (A.A.); anastasia.rigatou8@gmail.com (A.R.); 3 Athens Medical Center, 15125 Athens, Greece; valiadask@gmail.com; 4 Department of Cardiology, Veria General Hospital, 59132 Veria, Greece; evag.sdogkos@gmail.com; 5 Second Department of Critical Care, Attikon General University Hospital, National and Kapodistrian University of Athens, 12462 Athens, Greece; eikaras1@gmail.com; 6 Department of Biological Chemistry, Medical School, National and Kapodistrian University of Athens, 11527 Athens, Greece; madalamaga@med.uoa.gr

**Keywords:** sepsis-associated acute kidney injury, pathophysiology, diagnostics, RNA omics, human recombinant alkaline phosphatase, therapeutics

## Abstract

Sepsis-associated acute kidney injury (SA-AKI) is defined as the development of AKI in the context of a potentially life-threatening organ dysfunction attributed to an abnormal immune response to infection. SA-AKI has been associated with increased mortality when compared to sepsis or AKI alone. Therefore, its early recognition is of the utmost importance in terms of its morbidity and mortality rates. The aim of this review is to shed light on the pathophysiological pathways implicated in SA-AKI as well as its diagnostics and therapeutics. In this review, we will elucidate upon serum and urinary biomarkers, such as creatinine, cystatin, neutrophil gelatinase-associated lipocalin (NGAL), proenkephalin A 119–159, interleukin-6, interleukin-8 and interleukin-18, soluble toll-like receptor 2 (sTLR2), chemokine ligand 2 (CCL2) and chemokine C-C-motif 14 (CCL14). In addition, the role of RNA omics as well as machine learning programs for the timely diagnosis of SA-AKI will be further discussed. Moreover, regarding SA-AKI treatment, we will elaborate upon potential therapeutic agents that are being studied, based on the pathophysiology of SA-AKI, in humans and in animal models.

## 1. Introduction

According to the Third International Consensus Definitions for Sepsis, sepsis is a state of potentially life-threatening organ dysfunction, attributed to an abnormal immune response to infection [1]. This extreme response is driven by hyperinflammation and may lead to multiple organ dysfunction, septic shock or even death. From data published in 2020, it has been estimated that sepsis affects 48.9 million cases and 11 million sepsis-related deaths worldwide [2]. Despite the fact that these estimations are difficult to ascertain, it is assessed that approximately 20% of deaths have been associated with sepsis globally [2,3]. When considering the growing burden of sepsis alongside the escalating challenge of antimicrobial resistance, it becomes clear that sepsis represents a major public health concern.

Sepsis-associated acute kidney injury (SA-AKI) is defined as the development of AKI in the context of sepsis [4]. According to KDIGO (Kidney Disease Improving Global Outcomes), AKI is an increase in the levels of serum creatinine by 50% within 7 days or an increase in serum creatinine by 0.3 mg/dL within 2 days or a reduction in urine output within at least 6 h [4]. It has been estimated that among hospitalized critically ill patients with AKI, sepsis accounts for approximately 45% to 70% of all cases [4,5]. Mehta et al. in their meta-analysis have reported that longer duration of AKI, for more than seven days, has been associated with increased risk of long-term renal adverse effects, such as chronic kidney disease and end stage renal disease [6]. However, long-term renal sequelae after SA-AKI have not yet been established. This may be due to the very recent definitions of SA-AKI and the difficulties associated with the evaluation of the exact incidence of SA-AKI as well as the estimation of its long-term renal adverse effects. Nevertheless, it has been demonstrated that persistent AKI, as defined by the presence of AKI for >48 h in the context of sepsis, has been related to an increased 30-day and one-year mortality [7]. SA-AKI is divided into early SA-AKI, which is documented within 48 h of the onset of sepsis and late SA-AKI, and occurs between 48 h and 7 days of sepsis onset [8]. Regarding its severity, as it would be anticipated, SA-AKI has been estimated to result in increased mortality when compared to solely sepsis or AKI [9]. Therefore, its timely recognition remains of the utmost importance. The aim of this review is to elucidate upon various biomarkers that could aid the early recognition of SA-AKI. In order to accomplish a timely recognition of this clinical entity, we will refer to the pathogenesis of SA-AKI as well. In addition, we will thoroughly discuss the utility of each biomarker. Moreover, we will examine current therapeutic options as well as ongoing research regarding potential therapeutic drugs that should be further evaluated in large-scale studies in the near future.

## 2. Pathogenesis of SA-AKI

### 2.1. The Role of Inflammation

Inflammation plays a crucial role in sepsis and SA-AKI. Specifically, toll-like receptors (TLRs) comprise a family of pattern recognition receptors (PRRs), which are located mainly, but not solely, on immune cells and play a pivotal role in the so-called “cytokine storm”. When TLRs recognize microbial components known as microbial-associated molecular patterns (MAMPs) or damage-associated molecular patterns (DAMPs), activation of multiple pro-inflammatory pathways occurs, characterized by the excess production of chemokines, cytokines, and reactive oxygen species (ROS) [10,11,12,13]. Increased production of chemokines and cytokines stems from an attempt by the host to contain the spread of the microbe and to battle with it, in order to overcome this obstacle and, finally, to survive. However, when a cytokine storm occurs, i.e., when the reaction of the host is exaggerated and overwhelmed, it may result in organ dysfunction. SA-AKI is the renal component of this organ dysfunction. SA-AKI may be partly attributed to the release of interleukin (IL)-6, IL-8, and tumor necrosis factor alpha (TNF-α) in the kidneys after recognition of TLR-2 and TLR-4 by tubular epithelial cells (TECs) in the kidney [13,14].

### 2.2. The Role of Metabolic Reprogramming

Metabolic reprogramming refers to metabolic alterations that occur due to substantial changes in the cells’ environment and is a highly conserved evolutionary process [15]. It is an evolutionary mechanism protecting cells from an abrupt injury, such as hypoxia, and is meant to protect cells from death. This is accomplished by energy-saving processes, mainly the switch from oxidative phosphorylation (OXPHOS) to aerobic glycolysis. This metabolic switch is designed to alter energy production, prioritizing survival by reducing the reliance on OXPHOS and generating a more manageable supply of adenosine triphosphate (ATP) under abnormal or stress conditions.

Regarding SA-AKI, proximal TECs are the second most abundant in mitochondria cells, just after cardiomyocytes [16]. This fact underlies their increased demand for energy supplies. Therefore, in cases of sepsis, immune cells like monocytes and lymphocytes exhibit this metabolic switch from OXPHOS to aerobic glycolysis, a process known as the Warburg effect [17]. With this metabolic switch, monocytes and lymphocytes develop into a more favorable profile, especially M1 (M1 macrophages) and Th-17 (T helper-17) cells, thus producing pro-inflammatory cytokines [18,19,20]. Concerning TECs, this metabolic switch to aerobic glycolysis seems to be a “shut down model”, while re-prioritizing cells energy balance [15]. However, further research in the field of metabolic reprogramming in terms of TECs in sepsis is warranted.

### 2.3. The Role of Cell Death

Apoptosis, pyroptosis, necroptosis, ferroptosis, autophagy and efferocytosis are the main pathways driving cell death and removal of any unnecessary cellular components [10]. Apoptosis is a form of programmed cell death characterized by a reduction in cell volume due to membrane shrinkage that is mediated by an activation of caspases [21]. Pyroptosis is cell death that occurs as a result of activation of the inflammasome, especially the nucleotide binding domain-like receptor 3 (NLRP3). By means of the NLRP3, gasdermin-D, which is activated by caspase-1 in the canonical pathway of NLRP3, forms pores in the cellular membrane, leading to alterations in osmotic pressure and thereby cell death by pyroptosis [22,23,24,25]. Necroptosis is a form of cell death that is seemingly the opposite of apoptosis, as in necroptosis there is swelling of the organelles and not shrinkage of the cell membrane, but rupture of the cell membrane on account of the increased cell volume. Necroptosis is mediated by receptor interacting protein kinases (RIPK) 1 and 3 [10]. Ferroptosis is a relatively newly discovered form of cell death, in which iron ions are most likely implicated [26,27]. Iron ions’ accumulation in cells is proposed to result in the mitigation of cellular glutathione levels and, ultimately, in cell death [26,27,28,29].

Autophagy is a complex process characterized by the destruction of organelles and the removal of any unnecessary components of the cell in order to achieve homeostasis [30]. Autophagy is performed through fusion with the lysosomes to form the phagolysosome [30,31]. In this complex process, genes, mainly autophagy-related genes (*ATG*) *atg5* and *atg7*, together with the mammalian target of rapamycin (mTOR), the sirtuins and nuclear factor kappa-light-chain-enhancer of activated B cells (NF-κΒ) are intricately implicated [30,31]. Finally, efferocytosis is the process of removal of dead cells by “professional” macrophages, like dendritic cells and macrophages, and “non-professional” macrophages, such as fibroblasts, which target the “eat me” signals [32,33]. In this process, the apoptosis inhibitor of macrophages (AIM) is usually implicated by binding to immunoglobulin IgM. Regarding the kidney, AIM binds to kidney injury molecule-1 (KIM-1) and this binding further provokes SA-AKI [10].

### 2.4. The Role of Hemodynamic Changes

**Figure 1:**
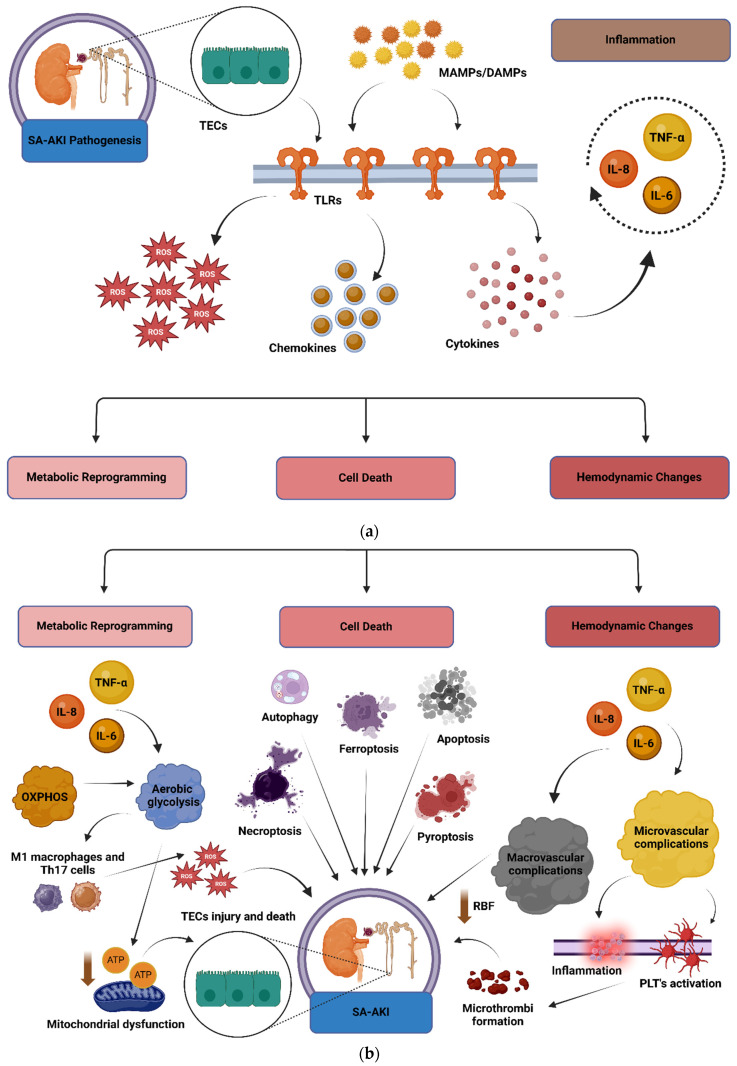
(**a**). The vital role of inflammation in the development of SA-AKI. The activation of TLRs on kidney TECs by MAMPs or DAMPs triggers the production of cytokines, chemokines, and ROS, initiating a systemic inflammatory response. This culminates in a cytokine storm, which promotes renal injury through the action of pro-inflammatory mediators such as IL-6, IL-8, and TNF-α, ultimately contributing to SA-AKI. Inflammation also influences metabolic reprogramming, driving a shift towards glycolysis, enhances cell death pathways such as apoptosis, necroptosis, and pyroptosis, and disrupts hemodynamic stability, including alterations in renal blood flow and microvascular function, which together exacerbate kidney injury [6,7,8,9,10,11]. Abbreviations: CCL-2: Chemokine C-C motif ligand 2; DAMPs: Damage-Associated Molecular Patterns; IL: Interleukin; MAMPs: Microbial-Associated Molecular Patterns; ROS: Reactive Oxygen Species; SA-AKI: Sepsis-Associated Acute Kidney Injury; TECs: Tubular Epithelial Cells; TLR: Toll-Like Receptor; TNF-α: Tumor Necrosis Factor-alpha. Created in BioRender. Kounatidis, D. (2024) https://BioRender.com/o88k763 (accessed on 8 December 2024). (**b**). Metabolic reprogramming, cell death, and hemodynamic alterations during SA-AKI. During sepsis, inflammatory cytokines (e.g., TNF-α, IL-6, IL-1β) are produced in response to infection and alter cellular metabolic pathways in immune cells and renal TECs. This inflammatory milieu induces a metabolic shift from OXPHOS to aerobic glycolysis, a process known as the Warburg effect, which supports rapid energy production in response to cellular stress. Activated immune cells, such as M1 macrophages and Th17 cells, heavily rely on aerobic glycolysis to meet the high energy demands of cytokine production and other immune functions. Glycolysis also promotes the production of ROS, which amplifies inflammation. TECs, which normally depend on mitochondrial OXPHOS for ATP production, experience mitochondrial dysfunction during inflammation. This dysfunction induces a metabolic switch to glycolysis to conserve energy under hypoxic and inflamed conditions. However, this shift leads to reduced ATP production, impairing cellular processes and repair mechanisms, ultimately resulting in TEC injury and death. While initially protective, prolonged metabolic reprogramming contributes to energy deficits and impaired repair, culminating in cell death, such as apoptosis and ferroptosis. Additionally, persistent inflammation and platelet activation cause macrovascular and microvascular alterations, including impaired renal blood flow and microthrombi formation, which exacerbate ischemia and worsen SA-AKI [10,11,12,13,14,15,16,17,18,19,20,21,22,23,24,25,26,27,28,29,30,31,32,33,34]. Abbreviations: ATP: Adenosine Triphosphate; IL: Interleukin; OXPHOS: Oxidative Phosphorylation; PLTs: Platelets; ROS: Reactive Oxygen Species; SA-AKI: Sepsis-Associated Acute Kidney Injury; TECs: Tubular Epithelial Cells; Th17: T helper 17; TNF-α: Tumor Necrosis Factor-alpha. Created in BioRender. Kounatidis, D. (2024) https://BioRender.com/i25a010 (accessed on 8 December 2024).

Macrovascular together with microvascular changes are suggested to be involved in the development of SA-AKI. More specifically, how to maintain normal renal blood flow (RBF) in sepsis still remains a subject of debate. However, in cases of septic shock, intravenous fluid administration and the use of vasopressors in order to maintain a mean arterial pressure (MAP) > 65 mmHg have been documented to increase urinary output [10]. Microvascular changes refer to the formation of microthrombi due to inflammatory alterations and platelet activation, which may result in disseminated intravascular coagulation (DIC) [10,34]. Figure 1a,b depict the role of inflammation, metabolic reprogramming, the various types of cell death, as well as the role of hemodynamic changes in the development of SA-AKI.

## 3. Diagnostics of SA-AKI

### 3.1. Serum Biomarkers for SA-AKI

There is ongoing research regarding the early diagnosis of SA-AKI. Since serum creatinine levels typically rise only after more than 50% of kidney function has already been lost, there is an urgent need for earlier and more precise diagnostic methods to detect kidney dysfunction in its initial stages. Thus, cystatin C and neutrophil gelatinase-associated lipocalin (NGAL) have emerged as promising biomarkers of AKI. Cystatin C is a cysteine protease inhibitor produced at a constant rate by all nucleated cells. It is a 13 kDa protein, which is freely filtered by the glomeruli, reabsorbed and catabolized in the proximal tubules [35]. Due to its aforementioned properties, it has been suggested to detect AKI much earlier than serum creatinine levels. Unlike serum creatinine levels, serum cystatin C levels are not significantly affected by parameters, such as muscle mass, age, gender, weight and height [36]. Cystatin C has already been incorporated into estimated glomerular filtration rate (eGFR) calculations, either as a standalone marker or in combination with serum creatinine levels [37,38].

NGAL is a 24 kDa glycoprotein secreted by the kidneys, especially by tubular cells in cases of AKI. It has originally been found on activated neutrophils, but nowadays, its secretion by tubular cells in the kidney has turned NGAL into a useful tool for measurement of AKI and chronic kidney diseases as well [38]. NGAL has been suggested to be increased 10 times in the blood and up to 100 times in the urine of patients with AKI [39]. Notably, urinary NGAL has very recently been demonstrated to outperform serum NGAL among patients with SA-AKI [40]. Blood NGAL has been associated with the inflammatory process and chemotaxis that occurs during sepsis. Therefore, it is elevated among patients with sepsis. However, as NGAL is reabsorbed in the renal tubules under normal circumstances, in cases of SA-AKI, urinary NGAL should be increased as well [40]. Notably, Hu et al. demonstrated that blood NGAL was increased among patients with sepsis, but not SA-AKI. Nevertheless, within 24 h of admission to the ICU department, patients with SA-AKI displayed increased urinary NGAL levels, unlike patients with sepsis, but without SA-AKI [41]. Therefore, they concluded that urinary NGAL has high sensitivity and specificity for SA-AKI [40].

Nevertheless, other serum biomarkers have also emerged in the field of diagnosing SA-AKI, such as proenkephalin A 119-159 (penKiD), Interleukin-6 (IL-6), IL-8, galectin-3, presepsin and sTLR2 (soluble TLR2) [14]. Proenkephalin A 119-159 belongs to the family of enkephalins, i.e., endogenous opioids that act by binding mainly to delta opioid receptors [42]. Apart from the central nervous system (CNS), the kidney is the second most abundant location of delta opioid receptors, where the enkephalins act [43]. Proenkephalin A 119-159 is not protein-bound and is filtered by the glomeruli, which makes it a fruitful surrogate of AKI, especially in the context of sepsis [44,45]. Indeed, penKiD has been documented to be strongly related to measured GFR, as calculated by the gold standard method using iohexol clearance among patients with septic shock [45]. Lately, it has been associated with a 28-day mortality rate among patients with sepsis in the ICU [45]. IL-6 and IL-8 have also been found to be increased in patients with SA-AKI, as they are well-known pro-inflammatory cytokines [4,46].

Furthermore, galectin-3 is a glycan-binding protein that has been proposed to be implicated in SA-AKI [47,48]. More specifically, galectin-3 has been shown to promote the uptake of lipopolysaccharides (LPS) by TLR4 by activating the caspase-4/11 pyroptotic pathway associated with SA-AKI [47]. In addition, presepsin is a molecule that is produced after cleavage of the CD14 LPS receptor, which is mainly expressed on macrophages and monocytes [49]. Presepsin is a relatively new biomarker of infection characterized by a short half-life and more rapid elevation in serum levels compared to procalcitonin or C-reactive protein (CRP) [49,50]. Additionally, presepsin has a low molecular weight of 13 kDa and is eliminated by the kidneys, making it a highly promising biomarker for the early detection and assessment of SA-AKI [49,50,51]. In a study involving 193 patients presenting with sepsis as defined by the SEPSIS-3 criteria in the emergency department, presepsin was shown to be a reliable predictive biomarker for SA-AKI [51].

Similarly, sTLR2 has recently emerged as a promising biomarker for predicting SA-AKI [52,53]. Specifically, a study of 116 intensive care unit (ICU)-admitted sepsis patients demonstrated that elevated serum sTLR2 levels in the early stages of sepsis were predictive of SA-AKI development [52]. Zhang et al. have proposed that the soluble urokinase-type plasminogen activator receptor (suPAR) could serve as a biomarker for SA-AKI. In their study of 179 sepsis patients, logistic regression analysis revealed that suPAR levels measured 24 h after sepsis diagnosis were predictive of SA-AKI development [54]. Additionally, as inflammation is central to sepsis and insulin resistance is considered an inflammatory process, Fang et al. hypothesized that the triglycerides–glucose (TyG) index might predict SA-AKI [55]. Their study, which included 1426 sepsis patients, found that 78.5% developed SA-AKI, and the TyG index (a marker of insulin resistance) was significantly associated with SA-AKI and hospital length of stay. Fang et al. emphasized the need for further research on the TyG index in the context of sepsis and SA-AKI [55].

### 3.2. Urine Biomarkers for SA-AKI

Aside from serum biomarkers, urinary biomarkers are also useful in the diagnosis of SA-AKI [46,56,57,58,59]. More specifically, urine output below 0.5 mL/Kg/hour for 6 h is the cut-off for reduced urine output as defined in the KDIGO guidelines [8]. In addition to urine output, dipstick albuminuria and urine microscopy are useful tools for assessing AKI. Furthermore, several urinary biomarkers, such as urinary NGAL (uNGAL), KIM-1, chemokine C-C-motif ligand 14 (CCL-14), soluble triggering receptor expressed by myeloid cells (sTREM), tissue inhibitor of metalloproteinases-2 insulin-like growth factor-binding protein 7 (TIMP-2) (IGFBP7), IL-18, CCL-2, and liver-type fatty acid binding protein (L-FABP), have all been proposed as early markers for SA-AKI [46,56,57,58,59]. However, while these biomarkers demonstrate high accuracy in detecting AKI, they lack specificity for SA-AKI. Notably, CCL-14 has recently been identified as outperforming other urinary biomarkers in the early prediction of AKI [38]. Additionally, urine CCL-2, also known as monocyte chemoattractant protein-1 (MCP-1), has shown promise as a marker for the early prediction of SA-AKI as well [59]. Peng et al. studied urine CCL-2 levels in 216 ICU patients and found that urine CCL-2 levels were able to differentiate between SA-AKI and non-sepsis-related AKI [59]. However, the issue of specificity for SA-AKI remains unresolved, particularly for urinary biomarkers of SA-AKI (Table 1).

### 3.3. Non-Coding RNAs in SA-AKI Diagnosis

Nowadays, with the advent of RNA omics, the contribution of non-coding RNAs, such as microRNAs (miRNAs), long non-coding RNAs (lnRNAs), circular RNAs (circRNAs), small interfering rRNAs (sirRNAs) and tRNAs seem to be very promising in the early diagnostics of SA-AKI [60,61,62]. Among these non-coding RNAs, circRNAs are suggested to play a pivotal role in diagnosing SA-AKI, as circRNAs are stable, highly conserved among different species and widely expressed in many cells. Moreover, they have a longer lifespan than linear RNAs and are resistant to RNase R, which renders them surrogates for molecular biomarkers [63,64,65,66]. Table 2 depicts major studies advocating circRNAs as well as miRNAs as potential biomarkers of SA-AKI [64,65,66,67,68,69,70,71,72].

### 3.4. Microbiome and Metabolomics in SA-AKI

In addition to serum and urinary biomarkers, as well as RNA omics, studies on the human microbiome have identified potential biomarkers for SA-AKI [73]. Xu et al., using the Kyoto Encyclopedia of Genes and Genomes (KEGG) database, highlighted significant differences in the metabolite N6-N6-N6 Trimethyl-L-Lysine between patients with SA-AKI and those without [74]. This metabolite’s lysine degradation pathway has also been linked to the citrate cycle (TCA), suggesting its relevance in SA-AKI pathophysiology. Xu et al. proposed that the lysine degradation pathway might serve as a source of serum and urinary metabolite biomarkers for SA-AKI [74].

Although their study included only four patients with SA-AKI and five without AKI, it underscores the potential of serum and urinary metabolomics in this context. Furthermore, as suggested by Yang et al., SA-AKI may alter the composition of the gut microbiota [75]. Xu et al. reported a decreased abundance of beneficial anaerobes, such as *Bifidobacterium* species, in patients with SA-AKI compared to those without AKI [74]. However, research into gut microbiota alterations and metabolomics in SA-AKI remains in its infancy. Continued investigations are essential to further elucidate metabolomics-based biomarkers and their role in SA-AKI.

### 3.5. Machine Learning Approaches in SA-AKI Diagnosis

In recent years, there has been increased interest in machine learning programs regarding the early recognition of SA-AKI [76,77,78,79,80]. Instead of relying on a single biomarker, machine learning programs integrate multiple parameters to predict the development of SA-AKI and assess mortality risk in sepsis. Given the multifactorial nature of SA-AKI, these advanced computational models show significant promise and have recently garnered considerable attention. Luo et al. among 12,132 patients with SA-AKI used the Extreme Gradient Boosting program (XGBoost) and demonstrated this model to be a promising one regarding mortality prediction from SA-AKI [76]. In a study by Li et al., 8129 patients with sepsis were enrolled, and it was reported that the XGBoost algorithm performed exceptionally well in predicting mortality risk associated with SA-AKI [77]. Gao et al. studied a machine learning model among 12,196 patients with sepsis and found that this model was highly predictive of mortality associated with SA-AKI [78]. Similarly, Shi et al., in a cohort of 10,575 sepsis patients, demonstrated that the Light Gradient Boosting Machine (Light GBM) algorithm had the highest diagnostic power in predicting the development of SA-AKI. Notably, Shi et al. compared several machine learning models, including Light GBM, Support Vector Machines (SVMs), Artificial Neural Networks (ANNs), Decision Trees (DTs), Random Forests (RFs), and XGBoost, highlighting the superior performance of Light GBM in this context [79]. Unlike Logistic Regression, which oversimplifies relationships by assuming linear regression, machine learning models can handle more complex, non-linear analyses. This is a key factor behind the enhanced diagnostic potential of machine learning models in predicting SA-AKI [80].

## 4. Therapeutics of SA-AKI

The therapeutic approach to SA-AKI follows general principles, including the early administration of appropriate antibiotics in optimal doses, fluid resuscitation, the use of vasoconstrictive agents to maintain a MAP > 65 mmHg, and the timely initiation of renal replacement therapy (RRT). Restoration of the intravascular volume that aims to improve renal blood flow, while avoiding fluid overload, is essential in this regard. Therefore, daily and an additional fluid assessments are highly recommended. In addition, early versus late SA-AKI are likely to designate different approaches. In early SA-AKI, maintaining hemodynamic stability with fluid administration may be more desirable, whereas in late SA-AKI, fluid overload management is a more frequent requirement [4]. On the other hand, initiation of RRT in SA-AKI is suggested to follow recommendations as in non-septic patients with AKI [4]. Despite the fact that the IDEAL-ICU study has shown no benefit for early RRT versus later initiation in terms of outcomes of SA-AKI, there is still much debate on this issue [4,81,82,83]. Nevertheless, clinical assessment remains the cornerstone of initiating RRT, while biomarkers may be helpful in guiding decisions in this regard, as differential effects have been reported [4,82,83]. It is noteworthy that Seymour et al. have reported on the existence of four different phenotypes among patients with sepsis. Phenotype α was associated with lower vasoconstriction administration; phenotype β with older age, comorbidities and more frequent renal disease; phenotype γ with pulmonary disease and more evident inflammation; and phenotype δ with liver disease and septic shock. These four phenotypes have shown differential effects regarding clinical outcomes and host–response patterns [84]. Lately, there are multiple phenotypes proposed with different pathophysiological mechanisms known as endotypes, which may respond differentially to various therapeutic agents [85,86]. Beyond the aforementioned fundamental measures, ongoing research is exploring treatments targeting the specific pathophysiological mechanisms of SA-AKI.

ATP has been identified as a “danger” molecule with pro-inflammatory properties in AKI [87]. In SA-AKI, ATP levels are elevated in the kidneys due to its release from damaged renal cells. Conversely, alkaline phosphatase (ALP) has demonstrated anti-inflammatory effects in sepsis and SA-AKI in animal models [88]. ALP’s exogenous administration in septic models has shown promise, likely due to its ability to dephosphorylate LPS from Gram-negative bacteria, thereby reducing LPS toxicity [88,89]. In human studies, recombinant ALP (ilofotase alfa) has shown protective effects against SA-AKI. A Phase 3 trial, the REVIVAL study, involving 649 patients, found that ilofotase alfa improved major adverse outcomes by day 90, although it did not significantly enhance survival rates [89]. A subsequent machine learning analysis of 570 patients from the study identified specific SA-AKI phenotypes that benefited most from ilofotase alfa, particularly patients with phenotype 2, characterized by more severe clinical conditions [89]. Ilofotase alfa’s reno-protective effects are attributed to its ability to metabolize ATP into adenosine, which interacts with adenosine receptors (A2ARs) and potentially other pathways, providing a dual mechanism of protection [87,88,89]. Further investigation into recombinant ALP is eagerly anticipated.

Emerging evidence suggests additional therapeutic agents may ameliorate SA-AKI, particularly in preclinical models [90,91,92,93,94]. For instance, resatorvid (TAK-242), a TLR4/NF-κB pathway inhibitor, has demonstrated efficacy in animal models of SA-AKI. As the TLR4/NF-κB pathway is critical to SA-AKI pathogenesis, resatorvid has been shown to improve mitochondrial function, reduce oxidative stress, and mitigate inflammatory responses in animal studies [90,91,92]. Another promising agent is melittin, a natural compound derived from bee venom. Melittin has been shown to inhibit ferroptosis via the nuclear factor erythroid 2-related factor 2/glutathione peroxidase 4 (NRF2/GPX4) pathway. NRF2, a transcription factor regulating antioxidant responses, enhances the expression of GPX4, which counters lipid peroxidation during ferroptosis [93]. Dodson et al. demonstrated that melittin activates NRF2 translocation, increasing GPX4 expression, thereby inhibiting ferroptosis and alleviating SA-AKI [94].

The Danlou tablet (DLT), a herbal preparation containing various plant extracts, has also shown anti-inflammatory effects in a mouse model of SA-AKI. According to Yu et al., DLT ameliorated SA-AKI by inhibiting the poly (ADP-ribose) polymerase-1/high-mobility group box 1 (PARP1/HMGB1) pathway, which plays a role in the inflammatory response [95,96,97]. In another preclinical study, Li et al. evaluated the aldose reductase inhibitor epalrestat in a rat model of SA-AKI. Epalrestat, known for its use in diabetic nephropathy, mitigated inflammation by inhibiting the protein kinase C (PKC)/NF-κB pathway, resulting in reduced expression of cytokines such as IL-6 and TNF-α and attenuated kidney damage [98]. Given its established use in diabetic patients, epalrestat may hold promise for human trials in SA-AKI.

**Figure 2:**
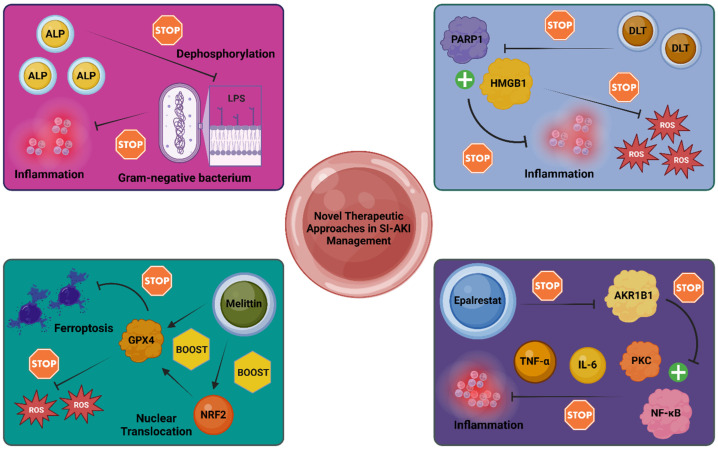
Emerging therapeutic interventions in SA-AKI: This figure depicts the mechanisms of action for four therapeutic agents in the context of SA-AKI. Recombinant ALP reduces the toxicity of LPS by dephosphorylation, thereby mitigating pro-inflammatory pathways. DLT, a herbal mixture, demonstrates anti-inflammatory and antioxidant effects, primarily through the inhibition of the PARP1/HMGB1 pathway. Similarly, epalrestat exerts anti-inflammatory effects by targeting the PKC/NF-κB pathway through AKR1B1 inhibition, leading to decreased IL-6 and TNF-α levels. Lastly, melittin enhances GPX4 expression via NRF2 nuclear translocation, diminishing ferroptosis, and oxidative stress [87,93,97,98]. Abbreviations: AKR1B1: Aldose Reductase; ALP: Alkaline Phosphatase; DLT: Danlou Tablet; GPX4: Glutathione Peroxidase 4; HMGB1: High-Mobility Group Box 1; IL-6: Interleukin-6; LPS: Lipopolysaccharide; NF-κB: Nuclear Factor kappa-light-chain-enhancer of Activated B cells; NRF2: Nuclear Factor Erythroid 2-Related Factor 2; PARP1: Poly (ADP-ribose) Polymerase 1; PKC: Protein Kinase C; ROS: Reactive Oxygen Species; SI-AKI: Sepsis-Associated Acute Kidney Injury; TNF-α: Tumor Necrosis Factor-alpha. Created in BioRender. Kounatidis, D. (2024) https://BioRender.com/e41h240 (accessed on 8 December 2024).

These advancements highlight the growing potential of targeted therapeutics in SA-AKI, emphasizing the need for further research to transition these findings into clinical practice. Figure 2 illustrates four distinct therapeutic approaches for ameliorating SA-AKI, as demonstrated by recent experimental studies.

## 5. Current Evidence and Future Perspectives

While general measures for the therapeutic management of SA-AKI are well-known and well-established, significant progress remains to be made in the fields of SA-AKI diagnostics and therapeutics. As the understanding of SA-AKI pathophysiology expands to encompass processes such as apoptosis, autophagy, necroptosis, pyroptosis, ferroptosis, efferocytosis, and metabolic reprogramming, the development of novel diagnostic biomarkers is highly anticipated. Recent discoveries of numerous serum and urinary biomarkers, alongside advancements in machine learning and multi-omics technologies, offer considerable promise. However, the integration of machine learning systems and multi-omics approaches remains cost-prohibitive at present. Despite these challenges, ongoing research into SA-AKI diagnostics and therapeutics holds great potential for yielding impactful advancements.

## 6. Conclusions

Despite the rapid expansion of research in the field of SA-AKI, diagnostic biomarkers and therapeutic interventions are still far from optimal. SA-AKI remains a significant consequence of sepsis, associated with a six- to eightfold increase in morbidity and mortality rates, underscoring the urgent need for advancements. While general therapeutic approaches provide a foundation, the development of more targeted and specific interventions is imperative to effectively and timely combat SA-AKI.

## Figures and Tables

**Table 1:** Summary of serum and urinary biomarkers of SA-AKI.

Serum Biomarkers of SA-AKI		
Creatinine [4,14]	Widely available and low cost. Necessary for eGFR calculation according to KDIGO.	It increases after severe kidney damage, i.e., when >50% of kidney function is already lost.
Cystatin C [35,36,37,38]	It is suggested to be less affected by factors, such as age, sex and muscle mass, unlike serum creatinine levels.	It is suggested to detect AKI earlier than serum creatinine. It has been embedded in various eGFR equations.
NGAL [38,39,40]	Produced by tubular cells as well as by neutrophils.	Increased in AKI/SA-AKI.
Proenkephalin A 119–159 [41,42,43,44,45]	It belongs to the family of enkephalins and the kidney is the second organ after the brain, where this molecule is abundant.	Increased in SA-AKI. Useful as a prognostic biomarker of SA-AKI.
IL-6 [4,46]	Inflammatory biomarker	Increased in SA-AKI.
IL-8 [4,46]	Inflammatory biomarker	Increased in SA-AKI.
Galectin-3 [47,48]	Inflammatory biomarker	Increased in sepsis/SA-AKI.
Presepsin [49,50,51]	Inflammatory biomarker	Increased approximately in 2 h in cases of bacterial infection. Early detection of sepsis.
sTLR2 [52,53]	Inflammatory biomarker	Early prediction of SA-AKI.
suPAR [54]	Inflammatory biomarker	Increased in AKI/SA-AKI.
TyG Index [55]	Triglycerides–glucose index is a marker of inflammation and insulin resistance.	Predictive of SA-AKI. Increased in sepsis and SA-AKI.
Urinary Biomarkers of SA-AKI		
Urine Output [8]	It is included in the KDIGO definition of AKI. Very useful.	The trajectory of urine output within 24 h of sepsis is highly predictive of SA-AKI development
Dipstick Albuminuria [4]	Rapid and low cost. It lacks specificity.	It provides general information regarding AKI.
Urine Microscopy [4]	Easy and widely available.	It provides general information regarding AKI.
uNGAL [38,39,40]	It is 10 times more increased in the urine than in the serum in cases of SA-AKI.	Increased in tubular damage/AKI.
IL-18 [56]	Inflammatory biomarker in urine.	Increased in SA-AKI
KIM-1 [4,46]	Early sensitive and specific urinary biomarker of AKI.	Increased in tubular damage/AKI.
CCL-14 [4,38]	Outperforms other urinary biomarkers for the early detection of AKI.	Increased in tubular damage/AKI. Predictive of persistent SA-AKI.
CCL-2 [59]	Inflammatory biomarker	Increased in SA-AKI.
L-FABP [4,56]	Early marker of AKI/SA-AKI	Increased in AKI/SA-AKI.
sTREM [4,46]	Early sepsis identification due to its increased production in sepsis.	Increased early is SA-AKI due to its higher production by renal resident cells.
TIMP2 * IGFBP7 [4,56,57,58]	Early prediction of SA-AKI.	Increased in SA-AKI with prognostic potential.

**Table 2:** Key studies highlighting circRNAs and miRNAs as potential biomarkers for SA-AKI.

Study/Year	Animal Model/ Population Studied	Findings	Remarks
Peng et al., 2024 [64]	8–10-week-old C57BL/6 male mouse kidney	Increased expression of mmu_Circ_26986 was associated with improvement of SA-AKI by means of the miRNA-29b-1-5p/PAK7 axis	mmu_Circ_26986/hsa_Circ_0072463 may be used as a potential target for SA-AKI
Li et al., 2024 [65]	8-week-old C57BL/6 male mouse kidney	miR-16-5p was increased in SA-AKI mouse models	miR-16-5 may be used as a biomarker of SA-AKI
Wang et al., 2023 [66]	C57BL/6 mouse kidney	Circ_0020339 was increased in SA-AKI mouse models	Circ_0020339 may serve as a biomarker of SA-AKI by targeting the miR17-5p/IPMK axis
Feng et al., 2023 [67]	10–12-week-old C57BL/6 male mouse	Circ_35953 sponged miR-7219-5p to modulate HOOK3 and IGFBP7 and may thus be a biomarker of SA-AKI	Circ_35953 mediates SA-AKI by targeting miR-7219-5pHOOK3 and IGFBP7 axis
Zhang et al., 2023 [68]	45 SA-AKI patients and 45 healthy controls	Circ_0114428 regulates miR-370-3p. TIMP2 is a target gene of miR-370-3p.	Circ_0114428 may serve as a biomarker of SA-AKI
Kuang et al., 2023 [69]	25 SA-AKI patients and 20 healthy controls	Circ_0001818 regulates miR-136-5p to modulate TXNIP/NLRP3 inflammasome pathway	Circ_0001818 may serve as a biomarker of SA-AKI
Zhou et al., 2023 [70]	19 SA-AKI patients and 17 healthy controls	Circ_0006944 via the miR-205-5p/UBL4 axis increases damage in SA-AKI	Circ_0006944 may be a biomarker of SA-AKI
Lu et al., 2022 [71]	23 SA-AKI patients and 23 healthy controls	Circ RNA HIPK3 sponged miR-338 to modulate FOXA1 axis	Circ RNA HIPK3 in increased in SA-AKI
Xu et al., 2022 [72]	SA-AKI patients and healthy controls	Circ_0114427 regulates miR-495-3pTRAF6 axis	Circ_0114427 is increased in SA-AKI and may serve as a biomarker of SA-AKI

Abbreviations: Circ_: Circular RNAs; FOXA1: Forkhead Box A1; hsa_Circ: Human circular RNA; HOOK3: Hook Microtubule Tethering Protein 3; IGFBP7: Insulin-Like Growth Factor-Binding Protein 7; IPMK: Inositol Polyphosphate Multikinase; miR-: MicroRNAs; mmu_Circ: Mouse circular RNA; NLRP3: NOD-Like Receptor Family Pyrin Domain Containing 3; PAK7: p21 (RAC1) Activated Kinase 7; SA-AKI: Sepsis-Associated Acute Kidney Injury; TIMP2: Tissue Inhibitor of Metalloproteinases 2; TRAF6: TNF Receptor-Associated Factor 6; TXNIP: Thioredoxin Interacting Protein; UBL4: Ubiquitin-Like Protein 4.

## Abbreviations

AKI: Acute Kidney Injury; AKR1B1: Aldose Reductase; ALP: Alkaline Phosphatase; ANN: Artificial Neural Networks; ATG: Autophagy Related Genes; ATP: Adenosine Triphosphate; CCL-2: C-C-Motif Ligand 2; CCL-14: C-C-Motif Ligand 14; CRP: C-Reactive Protein; CXCL: C-X-C Motif Chemokine Ligand; DAMPs: Damage-Associated Molecular Patterns; DIC: Disseminated Intravascular Coagulation; DLT: Danlou Tablet; DT: Decision Trees; eGFR: Estimated Glomerular Filtration Rate; FOXA1: Forkhead Box A1; GFR: Glomerular Filtration Rate; GPX4: Glutathione Peroxidase 4; HMGB1: High-Mobility Group Box 1; ICU: Intensive Care Unit; IGFBP7: Insulin-Like Growth Factor-Binding Protein 7; IL: Interleukin; KDIGO: Kidney Disease Improving Global Outcomes; KIM-1: Kidney Injury Molecule-1; L-FABP: Liver-Type Fatty Acid Binding Protein; LPS: Lipopolysaccharides; MAMPs: Microbial-Associated Molecular Patterns; MAP: Mean Arterial Pressure; MCP-1: Monocyte Chemoattractant Protein-1; mTOR: Mammalian Target of Rapamycin; NF-κB: Nuclear Factor Kappa-Light-Chain-Enhancer of Activated B Cells; NLRP3: Nucleotide Binding Domain Like Receptor 3; NRF2: Nuclear Factor Erythroid 2-Related Factor 2; OXPHOS: Oxidative Phosphorylation; PAK7: p21 (RAC1) Activated Kinase 7; PARP1: Poly (ADP-Ribose) Polymerase 1; PKC: Protein Kinase C; PRRs: Pattern Recognition Receptors; RBF: Renal Blood Flow; RIPK: Receptor Interacting Protein Kinases; ROS: Reactive Oxygen Species; RRT: Renal Replacement Therapy; SA-AKI: Sepsis-Associated Acute Kidney Injury; SIRT: Sirtuins; sTREM: Soluble Triggering Receptor Expressed by Myeloid Cells; suPAR: Soluble Urokinase-Type Plasminogen Activator Receptor; sTLR2: Soluble Toll-Like Receptor 2; SVM: Support Vector Machines; TCA: Tricarboxylic Acid Cycle; TGF-β: Transforming Growth Factor Beta; TLR: Toll-Like Receptor; TNF-α: Tumor Necrosis Factor Alpha; TRAF6: TNF Receptor-Associated Factor 6; TXNIP: Thioredoxin Interacting Protein; UBL4: Ubiquitin-Like Protein 4; XGBoost: Extreme Gradient Boosting.
